# Quasi-1D physics in metal-organic frameworks: MIL-47(V) from first principles

**DOI:** 10.3762/bjnano.5.184

**Published:** 2014-10-09

**Authors:** Danny E P Vanpoucke, Jan W Jaeken, Stijn De Baerdemacker, Kurt Lejaeghere, Veronique Van Speybroeck

**Affiliations:** 1 Center for Molecular Modeling, Ghent University, Technologiepark 903, Zwijnaarde 9052, Belgium

**Keywords:** band structure, density functional theory (DFT), low-dimensional electronics, metal-organic frameworks (MOFs), MIL-47

## Abstract

The geometric and electronic structure of the MIL-47(V) metal-organic framework (MOF) is investigated by using ab initio density functional theory (DFT) calculations. Special focus is placed on the relation between the spin configuration and the properties of the MOF. The ground state is found to be antiferromagnetic, with an equilibrium volume of 1554.70 Å^3^. The transition pressure of the pressure-induced large-pore-to-narrow-pore phase transition is calculated to be 82 MPa and 124 MPa for systems with ferromagnetic and antiferromagnetic chains, respectively. For a mixed system, the transition pressure is found to be a weighted average of the ferromagnetic and antiferromagnetic transition pressures. Mapping DFT energies onto a simple-spin Hamiltonian shows both the intra- and inter-chain coupling to be antiferromagnetic, with the latter coupling constant being two orders of magnitude smaller than the former, suggesting the MIL-47(V) to present quasi-1D behavior. The electronic structure of the different spin configurations is investigated and it shows that the band gap position varies strongly with the spin configuration. The valence and conduction bands show a clear V d-character. In addition, these bands are flat in directions orthogonal to VO_6_ chains, while showing dispersion along the the direction of the VO_6_ chains, similar as for other quasi-1D materials.

## Introduction

Metal-organic frameworks (MOFs) present a class of materials located at the conceptual interface between molecules and solids. They consist of inorganic metal or metal-oxide clusters (i.e., nodes) connected through organic molecules (i.e., linkers), giving rise to porous, highly tunable frameworks. Their porous nature, with internal surface areas of 1000 m^2^g^−1^ or more, and chemical tunability, through the choice of nodes and linkers, makes them versatile materials that are receiving an exponentially growing interest with a special focus on industrial, chemically oriented processes, such as catalysis, sensing, gas separation and gas storage [1–22].

In addition to providing large internal surface areas, the framework topology also allows to organize metal sites in a well-defined, ordered fashion, creating zero-, one- and two-dimensional metal(-oxide) structures. Such structures provide interesting systems to observe and study exotic and low-dimensional physics [23–37]. Transition-metal oxides, on the other hand, have proven to be a rich source of multiferroic materials [38–41]. Such materials, which combine at least two magnetic and/or electronic ordering phenomena, are of great interest for technological applications. MOFs containing transition-metal oxides as nodes are therefore expected to show physically interesting behavior. For example Canepa et al. [36] investigated the MOF-74 frameworks with Fe, Ni and Co at their metal centers, and found quasi-1D ferromagnetic behavior with quenched antiferromagnetic inter-chain interactions. Stroppa et al. [40] and Wang et al. [41] investigated Cu-based MOFs with an ABX_3_ perovskite architecture and found these to exhibit quasi-1D multiferroic behavior. In both cases, Jahn–Teller distortions of the Cu-ion environment were shown to play a crucial role in the 1D nature of the magnetic behavior. Chen et al. [37] reported on the observation of spin canting in a 2D Mn-based MOF with a transition temperature of 40 K and Sibille et al. [42] investigated the magnetism of the 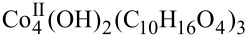 MOF. In each of these cases, a fundamental understanding of the electronic and magnetic properties was obtained by means of high-quality ab initio methods.

**Figure 1:**
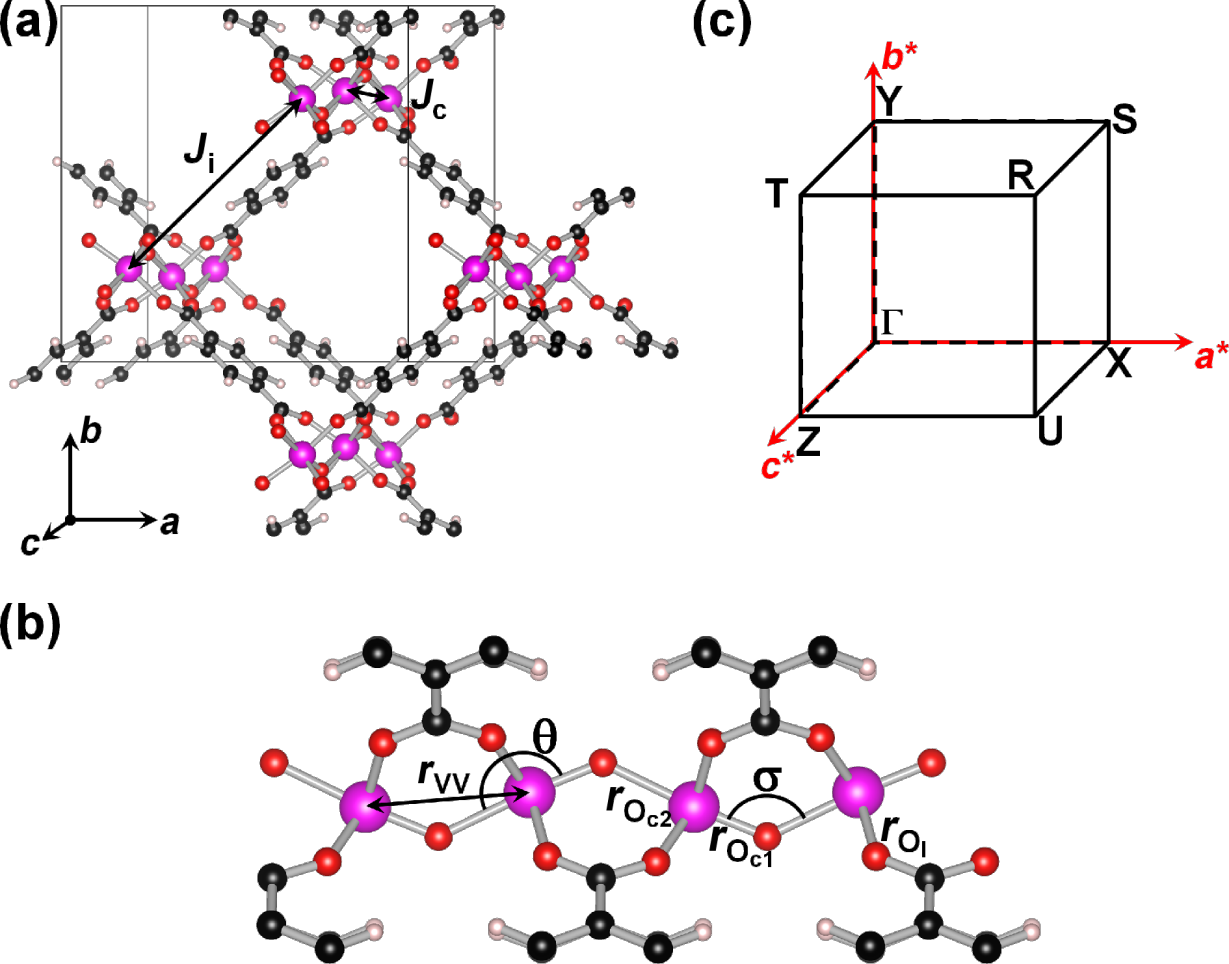
(a) Ball-and-stick representation of the MIL-47(V) MOF. Pink, red, black, and white spheres indicate V, O, C and H positions, respectively. The grey box indicates the unit cell used. The intra- and inter-chain couplings *J*_c_ and *J*_i_ (cf. section “Stability and magnetic coupling”) are indicated. (b) Representation of the Brillouin zone of the unit cell, showing the high symmetry *k*-points. (c) Ball-and-stick representation of a single vanadium oxide chain, indicating the superexchange angle σ, the octahedral backbone angle θ, the inter-V-distance *r*_VV_, and the V–O bond lengths 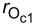 and 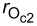 along the chain, and 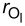 to the linker.

In this work, we present an ab initio investigation of the MIL-47(V) MOF [1] (cf. Figure 1a). The were three reasons to chose this particular MOF: (1) The topology of MIL-47(V) provides access to 1D metal-oxide chains. (2) The V version provides one unpaired electron per metal site, which is of interest for magnetic properties. (3) MIL-47 belongs to the family of so-called breathing MOFs [4,11,43–49], leading to interesting opportunities with regard to sensing applications. In this family, MIL-47(V) has a somewhat special status, because, unlike most breathing MOFs MIL-47(V^IV^) does not show breathing under thermal stimuli or after the adsorption of gases or liquids [5,44,50], but only under significant mechanical pressure [45]. In contrast, MIL-47(V^III^), also referred to as MIL-53(V), shows breathing behavior induced by temperature or by gas adsorption [44]. For the MIL-53(V) MOF, the presence of small amounts of V^IV^ has a detrimental effect on its flexibility, which indicates that the metal center plays an important role [44].

Because of the rigid nature of MIL-47(V^IV^) under standard breathing conditions it is often used as a material for comparison in studies of breathing (due to sorption) of other MOFs [3,7,10,13,51–52]. In addition, the 1D pores of MIL-47(V) make this material well-suited for gas storage and separation. As a result, much of the work since its first synthesis focuses on these topics. The adsorption and diffusion behavior of different molecules, ranging from hydrogen and carbon dioxide to methane and xylene isomers, has been studied both experimentally and theoretically [3,5–7,10,12–13,18–22,50–51]. The size of the MIL-47 system, however, limits the computational possibilities. As a result, most theoretical work in the above studies is limited to force-field based simulations [7,10,12–13,18–20,22,51]. In these, DFT calculations are often used to provide partial charges. Due to their computational cost (the work presented in this paper amounts to 25 years of CPU time), DFT calculations for other purposes tend to be limited to fixed geometries [50] or small *k*-point sets [53], with some exceptions [22].

In this paper, the influence of the spin configuration on the geometric and electronic structure is investigated: equilibrium structure, energy, bulk modulus and band structure. Also the transition pressure for the large-pore-to-narrow-pore phase transition is estimated, and inter- and intra-chain coupling constants are calculated.

## Computational details

### Density functional theory calculations

Density functional theory (DFT) calculations are performed within the projector augmented wave (PAW) method as implemented in the “Vienna ab initio Simulation Package” (VASP) while using the generalized gradient approximation (GGA) functional as constructed by Perdew, Burke and Ernzerhof (PBE) [54–58]. The plane wave kinetic energy cutoff is set to 500 eV. Due to the large difference in lattice vector lengths for the structures (cf. Figure 1) a Monkhorst–Pack special *k*-point grid of 2 × 2 × 6 *k*-points is used to sample the Brillouin zone [59–60]. Dispersive interactions, which play an important role in the flexibility of the crystal structure of MOFs [61], are included through the DFT-D3 method as formulated by Grimme et al. [62–63], including Becke–Johnson damping [64].

Due to the presence of Pulay stresses [65], MIL-47(V) tends to collapse during geometry optimization [60]. To prevent such collapse, the volume is optimized through fitting constant-volume optimized structures to the Rose–Vinet equation of state [60,66]. The constant-volume optimizations are performed by using a conjugate gradient method, allowing simultaneous optimization of atomic positions and cell shape. The convergence criterion is set to a difference in energy of less than 1.0 × 10^−7^ eV between subsequent ionic steps. After full relaxation, the forces on the ions are then found to be below 1.2 meV/Å.

The density of states (DOS) was obtained by using a denser *k*-point grid of 3 × 3 × 9 *k*-points, and the band structure was calculated along the edges of the first Brillouin zone (cf. Figure 1b).

The atomic charges in the systems are calculated by using the Hirshfeld-I approach [67–68] as implemented in our in-house-developed code HIVE [69–71]. The atom-centered spherical integrations [72] are done by using Lebedev–Laikov grids [73] of 1202 grid points per shell, and a logarithmic radial grid. The iterative scheme is considered to be converged when the largest difference in charge of a system atom is less than 1.0 × 10^−5^*e* between two consecutive iterations.

### Structure of MIL-47(V)

**Figure 2:**
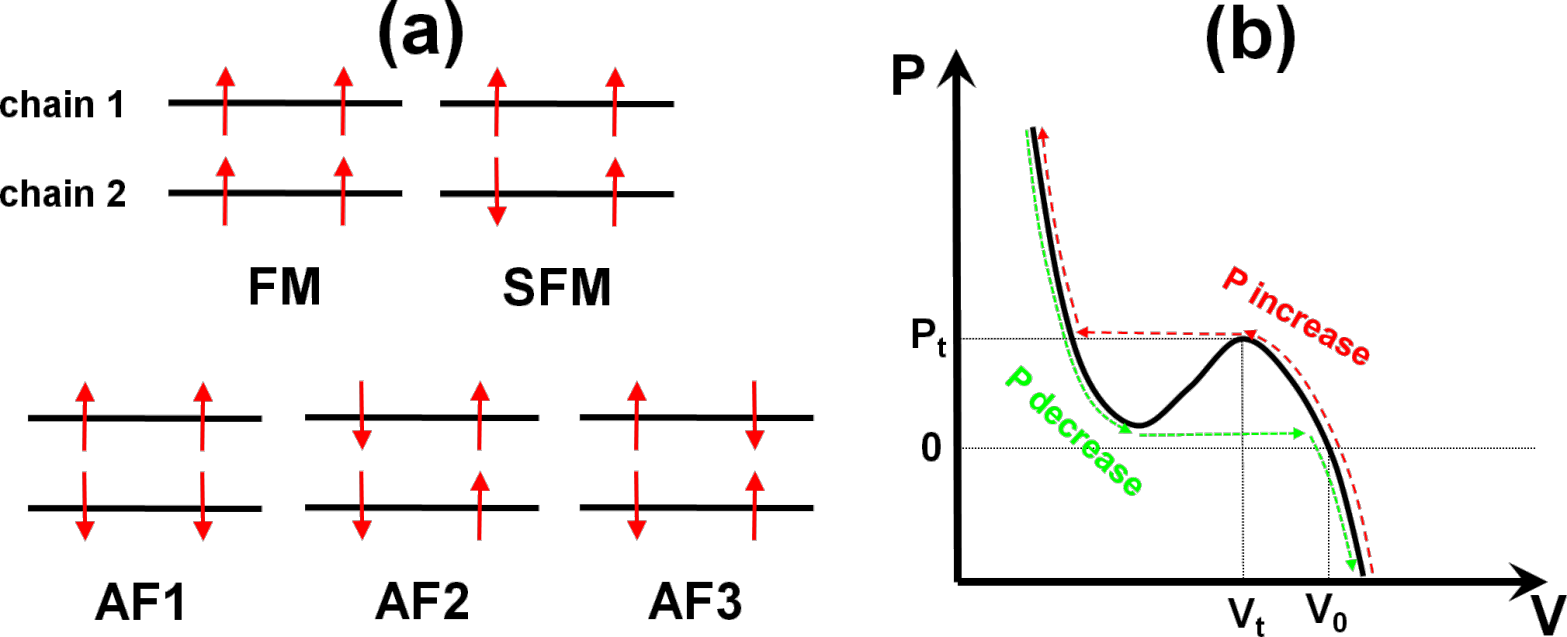
(a) Schematic representation of the five inequivalent magnetic configurations investigated in this work: ferromagnetic (FM), semi-ferromagnetic (SFM) with one ferromagnetic and one antiferromagnetic chain, and three different antiferromagnetic (AF) configurations. (b) Schematic representation of the P(V) relation of the MIL-47(V) MOF. The extrema of the s-shaped curve represent the points at which a pressure-induced phase transition occurs. The red dashed and green dotted curves indicate the path followed for increasing and decreasing pressure, respectively.

The periodic cell used in this work contains 4 formula units or 72 atoms, and is shown in Figure 1a. This cell contains 2 vanadium oxide chains with 2 vanadium atoms per chain. Each V atom contains one unpaired d-electron, since the V atoms have a formal charge of +IV in the MIL-47(V) topology. This leads to 2^4^ possible spin configurations of which five are inequivalent (cf. Figure 2): (FM) ferromagnetic for both inter- and intra-chain spin alignment; (SFM) semi-ferromagnetic, containing one ferromagnetic and one antiferromagnetic chain; (AF1) although globally antiferromagnetic, this system contains ferromagnetic chains in an antiferromagnetic configuration; (AF2 and AF3) systems containing antiferromagnetic chains in either a ferromagnetic (AF2) or antiferromagnetic (AF3) configuration.

## Results and Discussion

### Structure and elastic behavior

The optimized parameters of the Rose–Vinet equation of state [66] are presented in Table 1. The equilibrium volumes for the five different spin configurations are within a range of 2 Å^3^, while the lattice parameters are within a range of 0.4, 0.2, and 0.03 Å for the *a*-, *b*-, and *c*-axis, respectively. A comparison to the experimentally measured lattice parameters and volumes shows that the calculated values are generally slightly larger [1,6,22,44] (cf. Table 2), as it is expected from the under-binding nature of the PBE functional [74–77]. The calculated equilibrium volume of about 1555 Å^3^ is 1.3 and 1.7% larger than the experimental value measured by Barthelet et al. [1] and Leclerc et al. [44], respectively. Table 2 shows that the largest contribution to this deviation originates from the long *a*-axis (up to 1.6%), while the *b*-and the *c*-axis show a deviation from experiment of 1% or less. An interesting global aspect to note regarding the MIL-47(V) structure is the symmetry breaking in the V-chain. Whereas for the as-synthesized version all V atoms are positioned on a straight line, the V atoms exhibit a zig-zag configuration in the *b*–*c*-plane of the calculated structure. Although the separation in the *b*-direction (*b*_VV_) is rather small in absolute value (cf. Table 2), the resulting improvement in energy due to this symmetry breaking is about 300 meV for the 72-atom unit cell used. A more detailed investigation of the crystal geometry, presented in Table 2, shows that the vanadium–oxygen chains present almost identical bond lengths and angles, all in excellent agreement with the experimental values for such chains [1,6,78]. As a result, the different spin configurations give rise to nearly indistinguishable crystal structures. It is, however, important to note that these very small differences in the crystal structure do give rise to small variations in the system energy, in addition to the variations due to the different spin configurations. These small geometry contributions are not negligible, and lead to significant variations in the calculated coupling constants as will be shown in the following section (cf. section “Stability and magnetic coupling” and Table 3).

**Table 1:** Equilibrium structure parameters obtained from a 9-point fit to the Rose–Vinet equation of state with volumes ranging over ±4% with regard to the equilibrium volume: the ground state energy of the magnetic configuration relative to the ferromagnetic configuration (*E*_0_), the equilibrium volume (*V*_0_), the bulk modulus (*B*_0_) and its pressure derivative (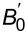). The root-mean-square deviation (rmsd) for each of the five fits is less than 0.8 meV. In addition, also the transition pressure *P_t_* and volume *V_t_* for which a large-pore MIL-47(V) is expected to transform into a narrow-pore structure is given.

	*E*_0_ [meV]	*V*_0_ [Å^3^]	*B*_0_ [GPa]	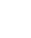 [–]	*P_t_* [MPa]	*V_t_* [Å^3^]

FM	0	1553.38	5.95	−53.2	83	1495.30
SFM	−144	1555.02	7.17	−52.2	102	1495.46
AF1	−16	1554.45	6.14	−55.6	82	1498.50
AF2	−279	1554.71	8.13	−48.5	124	1490.70
AF3	−278	1554.70	8.12	−48.5	124	1490.70

**Table 2:** Structural parameters of the equilibrium volume-optimized structures. *a, b*, and *c*: lattice parameters; 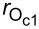, 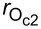, 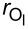: vanadium oxide bond length; *r*_VV_: intra-chain vanadium distance; σ: superexchange angle and θ octahedral backbone angle; *b*_VV_: component along the *b*-direction of *r*_VV_. Experimental data are given in comparison.

	*a* [Å]	*b* [Å]	*c* [Å]	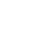 [Å]	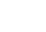 [Å]	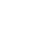 [Å]	*r*_VV_ [Å]	σ [°]	θ [°]	*b*_VV_ [Å]

FM	16.408	13.836	6.842	1.657	2.085	2.007	3.435	132.95	175.31	0.311
SFM	16.311	13.914	6.851	1.654	2.095	1.991	3.439	132.70	175.81	0.309/0.302^a^
AF1	16.397	13.844	6.847	1.656	2.087	2.005	3.437	133.00	175.36	0.311
AF2	16.237	13.969	6.855	1.654	2.097	1.991	3.441	132.69	175.88	0.302
AF3	16.231	13.975	6.854	1.654	2.097	1.975	3.440	132.69	175.87	0.301

MIL-47 experimentally derived structure										

exp. [1]	16.143	13.939	6.818	1.672	2.108	1.970	3.422	129.4	176.10	0.302
exp.^b^ [1]	17.519	12.168	6.875	1.947	1.947	1.995	3.438	123.98	180.00	0.000
exp.^c^ [6]	16.062	13.991	6.808	1.671	2.108	1.968	3.418	129.17	176.10	0.303
exp. [44]	16.070	13.960	6.818	–	–	–	–	–	–	–
exp. [22]	17.434	13.433	6.620	–	–	–	–	–	–	–

vanadyl acetate										

exp. [78]	14.065	6.877	6.926	1.684	2.131	2.002	3.480	131.2	174.6	–

^a^ferromagnetic/anti-ferromagnetic chain. ^b^MIL-47(V) as-synthesized. ^c^MIL-47(V) loaded with *meta*-xylene.

Table 2 shows that the VO_6_ octahedra are asymmetrically distorted. The double bond at one apex (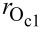 = 1.65 Å) lies in the center of the range of lengths of normal V=O bonds (1.55–1.75 Å), while the V···O *trans* bond (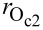) is at the lower end of the length range of such bonds (2.1–2.6 Å) [78]. The four bonds forming the plane of the octahedron (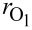) are about 0.1 Å shorter than the 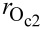 bond, which are typical single V–O bond lengths. A further distortion of the octahedral configuration is found in the O=V···O angle (the octahedral backbone angle θ), which is about 5° smaller than the expected 180°, showing the octahedra to bend toward the central axis of the chain. The alternating bridge position of the organic linkers leads to the undulate nature of the chains, giving rise to a superexchange angle σ of about 133° (cf. section “Atomic charges and magnetization”). These two angles show how the competition between the linker bridges and the V=O···V bridges affects the orientation of the VO_6_ octahedra in the chains; longer bond lengths (or weaker bonds) in the linker bridges will give rise to larger superexchange angles, changing the preference from antiferromagnetic to ferromagnetic interactions according to Goodenough rules [79].

Focusing on the local environment of the vanadyl chain in the MIL-47(V) MOF, one may wonder how strongly the nature of the linker influences the chain geometry. Removing the central four C atoms from the benzene ring, and protonating the dangling bonds of the remaining two C atoms, presents a system of vanadyl acetate chains, which are known to form in solvothermal reactions [78]. X-ray powder diffraction (XRPD) experiments (cf. Table 2) show these chains to have the exact same structure, suggesting that only the bridging part of the linker is of importance for the chain geometry.

In contrast to the structure parameters, the bulk modulus *B*_0_ displays a clear variation with the spin configurations (cf. Table 1), starting at about 6 GPa for structures presenting ferromagnetic chains (FM and AF1) up to 8 GPa for structures containing antiferromagnetic chains (AF2 and AF3). These values are in agreement with the elastic parameters calculated by Ortiz et al. [80–81] for MIL-47, and of the same order of magnitude found for other MOFs [82–83]. The pressure derivative 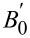 shows the same trend, i.e., it becomes larger (less negative) going from the ferromagnetic to the antiferromagnetic chains. Since the bulk modulus of a material is a measure for its resistance to deformation under an external pressure, and a negative pressure derivative (cf. 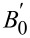 in Table 1) indicates a breakdown of this resistance under an applied pressure, a qualitative picture emerges in which the MIL-47(V) is expected to collapse or show a structural phase transition under sufficiently large external pressure. As with other MOFs of this topology, which are known as breathing MOFs, these results suggest that the MIL-47(V) MOF should show breathing behavior. However, in this case the breathing is due to the application of an external pressure. This qualitative picture is in good agreement with recent experimental observations by Yot et al. [45]

Based on the experimental observation of hysteresis in the *P*(*V*)-behavior of the MIL-47(V) MOF [45], we know that the *P*(*V*) relation should present an s-shape with a maximum at the large-pore-to-narrow-pore phase transition, and a minimum at the narrow-pore-to-large-pore phase transition (cf. Figure 2). By using the Rose–Vinet equation of state to generate the *P*(*V*) relation of the large-pore MIL-47(V) MOF, the large-pore-to-narrow-pore transition pressure *P*_t_ and transition volume *V*_t_ are calculated for each of the five spin configurations (cf. Table 1). Note that for the narrow-pore-to-large-pore transition, an equation of states fit to narrow-pore structures would be required, which is beyond the scope of this work. Relating *P*_t_ to the spin configurations yields two interesting features: (i) ferromagnetic chains (FM and AF1) give rise to a significantly lower transition pressure than antiferromagnetic chains (AF2 and AF3), (ii) the transition pressure for a system containing both types of chains (SFM) is a (weighted) average of the transition pressures of the antiferromagnetic and ferromagnetic systems. This provides interesting opportunities for sensor applications, e.g., combined with guest-induced magnetic transitions [35].

The calculated transition pressures are in good agreement with previous force-field based molecular dynamics simulations, which found *P*_t_ = 137 MPa [45]. In contrast to these simulations, experimental Hg-intrusion measurements did not give a transition at one specific pressure. Instead, the transition spanned a broader range of pressures: *P*_t_ = 85–125 MPa [45]. This is in perfect agreement with our calculations and suggests the sample consists of grains with varying mixtures of ferromagnetic and antiferromagnetic chains: Systems that only contain ferromagnetic chains show a phase-transition from large pores to narrow pores already at 82 MPa, while mixed systems with ever larger fractions of antiferromagnetic chains show increasingly higher transition pressures, until the systems contain only antiferromagnetic chains, which have the highest transition pressure of 124 MPa. Alternately, in XRPD experiments at room temperature while using a diamond anvil cell the transition pressure range was found to begin at 178.1 MPa [45]. At that pressure, the XRPD experiments discerned two phases of which the large-pore phase had a unit cell volume of 1506.6 Å^3^, which is in good agreement with the large-pore-form volumes *V*_t_ calculated in Table 1.

The differences in experimentally measured transition pressures were assigned to differences between the samples and the experimental conditions. In addition, it was suggested, based on XRPD, that different grain sizes may have different transition pressures, leading to a gradual transition of the entire sample [45].

In conclusion, the dependency of *P*_t_ on the spin configuration may provide insight in the relation between the ground state and the grain size. It is well-known that defects in a solid, such as grain boundaries, promote the presence of non-ground-state (sub)structures. When grains are, therefore, considered to consist of an internal bulk region surrounded by a surface shell region, it is natural for the MIL-47(V) MOF to assume that the internal region should (at low temperature) contain mainly antiferromagnetic chains (i.e., the ground state configuration). In contrast, ferromagnetic chains may dominate the surface region. In such case, smaller grains may have a larger ferromagnetic contribution, while large grains have a larger antiferromagnetic contribution. This might explain the experimentally observed range of *P*_t_. However, additional theoretical and experimental studies are required to formulate a definite conclusion in this regard.

### Stability and magnetic coupling

The spin configuration plays an important role in the stability of the system as is shown in Table 1. As is expected from magnetic measurements on MIL-47(V) [1] and magnetic susceptibility measurements on vanadyl acetate chains [78], an antiferromagnetic ground state is found, which is 70 meV per V atom more stable than the ferromagnetic state. Also note that the antiferromagnetic coupling of ferromagnetic chains (AF1) leads to a small improvement of the stability by 4 meV per V atom, showing that in addition to the intra-chain coupling of the V spin, an (albeit much weaker) inter-chain coupling is present as well.

To calculate the coupling interactions we have mapped the DFT energies onto a 1D Ising model:

[1]
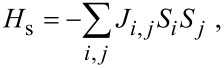


with *S_i_* being the spin operator projected along the *z*-direction and *J_i_*_,_*_j_* being the coupling interactions. Two coupling interactions are taken into account: the intra-chain coupling *J*_c_, and the inter-chain coupling *J*_i_ (cf. Figure 1a). The V magnetic moment in the current systems can be obtained by projection of the electron density onto atomic orbitals. However, in such an approach the magnitude of the obtained moment will strongly depend on the projection operation. As such, we will take a pragmatic stance and use spin 1/2 based on the presence of a single unpaired electron for each of the V ions, and the observed spin 1/2 for vanadyl acetate chains [78].

Each of the five spin configurations gives rise to a slightly different eigenvalue of the spin-Hamiltonian *H*_s_. By solving the overdetermined system of five equations (one for each configuration) using a least-squares fit, the coupling constants *J*_c_ and *J*_i_ are calculated. Table 3 shows both couplings to be antiferromagnetic in nature, with the inter-chain coupling being two orders of magnitude smaller than the intra-chain coupling. As a result, one expects the MIL-47(V) system to present (quasi-)1D behavior at low temperatures. The ratio of the coupling constants is much larger than those found for other MOFs (a factor of 5 was found for Cu-based perovskite MOFs [40], a factor of about 20 was found for MOF-74(X) with X = Co, Fe, Ni [36]). Our findings corroborate the suggestion of Barthelet et al. [1] that the antiferromagnetic behavior of the MIL-47(V) system stems from antiferromagnetic chains and not from antiferromagnetically ordered ferromagnetic chains. As a result, this also shows that the calculated superexchange angle of 133° is below the blank angle.

**Table 3:** Calculated coupling constants (in meV) based on the DFT-D3 energies by using the fully optimized geometries (DFT-D3), the pure DFT energies without D3 correction (DFT), and the DFT energies obtained by using the fixed AF3 ground-state atomic structure (DFT fix).

	DFT-D3	DFT	DFT fix

*J*_c_	−135.28	−131.81	−144.57
*J*_i_	−1.85	−1.59	−2.30

The coupling constants shown in Table 3 are rather large (|*J*_c_/*k*_B_| ≈ 1530–1678 K), in contrast to the values suggested from experiments: |*J*_c_/*k*_B_| = 275 K for vanadyl acetate chains [78], and |*J*_c_/*k*_B_| ≈ 186 K for MIL-47(V) [1]. This difference may have several reasons: (1) the experimental coupling constant is obtained from fitting a Curie–Weiss law to the linear high-temperature part of reciprocal magnetic susceptibility; (2) the choice of the DFT functional, e.g., based on LDA energies, not shown, the coupling constants are almost a factor of two larger. Also, Wang et al. [41] showed that the choice of the Hubbard U, in DFT+U calculations, significantly modifies the coupling constants.); (3) finite size effects (the calculated systems represent perfect infinite-size systems, while it was shown, for example, for vanadyl acetate chains that finite size contributions to the magnetization curve are significant [78]), and (4) the actual atomic structure used: Table 3 shows that the *J*_c_ coupling constant is about 10% smaller for geometries that are optimized while including the spin configuration. This effect is even more pronounced for smaller coupling constants. This last point may also be important for other systems in which energy differences can be even smaller, which is often the case for quasi-1D spin configurations in MOFs.

### Atomic charges and magnetization

**Figure 3:**
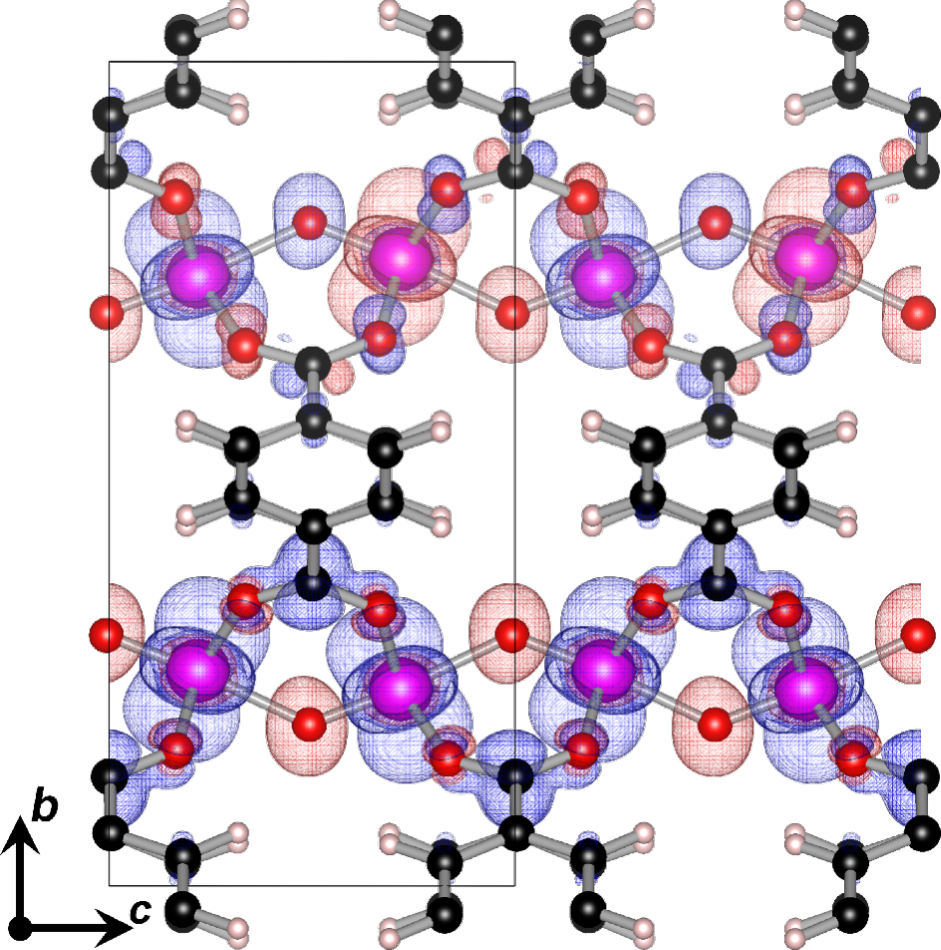
The spin density distribution of the SFM system. The upper chain has an antiferromagnetic spin configuration, while the lower chain has a ferromagnetic spin configuration. The iso-surface is taken at 0.00125, with majority spin shown in blue, and minority in red. The black rectangle indicates a single unit cell.

Hirshfeld-I (HI) atoms-in-molecules (AIM) charges [67,69–71] have been calculated to provide a better understanding of the superexchange mechanism in the vanadium oxide chains and the influence of the spin configuration on the electron distribution. For all spin configurations, the calculated V charge is found to be 2.44*e* and 2.43*e* for antiferromagnetic and ferromagnetic chains, respectively. This shows that the same oxidation state is present in both cases. Comparison to V charges in MIL-47(V) MOFs with functionalized linkers shows exactly the same charge, indicative of a +IV oxidation state [22]. Note that, as is to be expected from Hirshfeld-I charges, these charges are significantly larger than Mulliken (1.207*e*) [51] or CHELPG (1.68*e*) [12] charges. Similarly, the O atoms in the ferromagnetic chains have a slightly larger negative charge (−1.01*e*) than their counterparts in the antiferromagnetic chains (−1.00*e*). In contrast, the O atoms in the plane of the VO_6_ octahedra have an atomic charge of −(0.73 ± 0.01)*e* in line with their different bonding to the V atoms (cf. section “Structure and elastic behavior”). The same trends are also present in the calculated magnetization. For the V sites the magnetization is found to be slightly larger in ferromagnetic chains (≈0.9 μ_B_) than in the antiferromagnetic chains (≈0.8 μ_B_). However, both are indicative of a V^4+^ oxidation state. Interestingly, all O atoms in the vanadyl chains also show a small magnetization (0.06 and 0.08 μ_B_ in the antiferromagnetic and ferromagnetic chain, respectively) with a sign opposite to that of the magnetization of the nearest V atom (cf. Figure 3). Furthermore, also the in-plane O atoms present an even smaller opposing magnetization to the nearby V atom. From Figure 3 it is clear that the magnetization is localized (almost) entirely on the metal-oxide chains, in agreement with the relatively small inter-chain coupling. The induced magnetization supports the suggested presence of a superexchange mechanism. The larger charge and magnetization on the vanadyl O atoms presents a magnetic interaction pathway directly along the chain. Furthermore, the tilted nature of the magnetic orbitals increases their overlap, strengthening the antiferromagnetic interactions.

### Electronic structure

**Figure 4:**
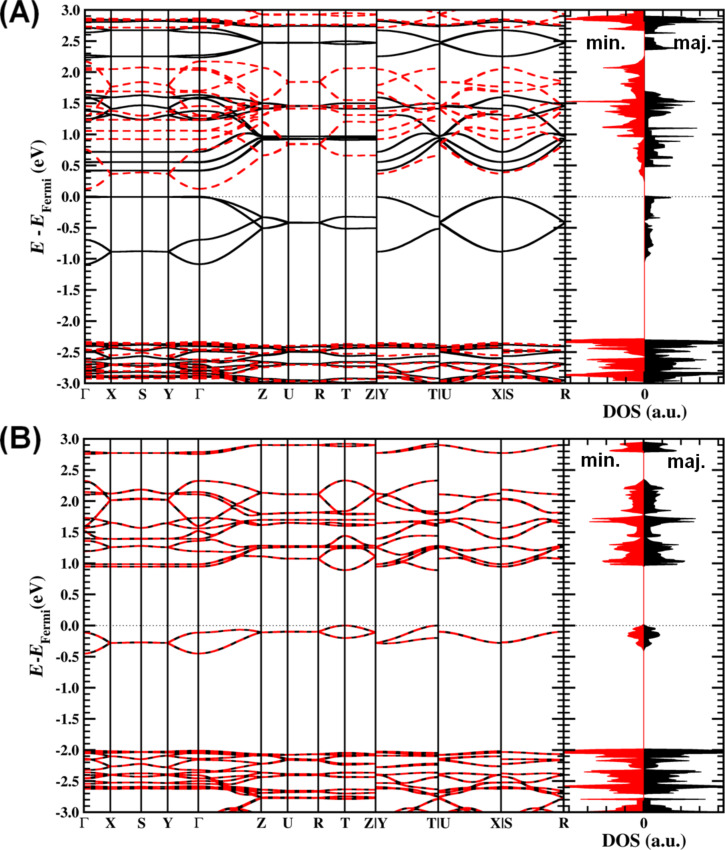
Band structure and density of states (DOS) near the Fermi level for the FM (A) and AF3 (B) spin configurations. Solid black/dashed red curves indicate the bands for the majority/minority spin components, respectively.

The MIL-47(V) systems show a very rich band structure around the band gap due to the interaction of the unpaired V d-electrons. The high-symmetry lines of the first Brillouin zone of the orthorhombic MIL-47(V) cell used are shown in Figure 1b. For the configurations for which the total magnetization is non-zero (FM and SFM), majority and minority spin components have a different band gap, making them of interest for spintronic applications (cf. Table 4 and Figure 4a) [84]. In general, each of the configurations leads to at least one direct band gap, which is located at a different point of high symmetry (cf. Table 4). The electronic structures for the FM and AF3 configurations are shown as examples in Figure 4.

**Table 4:** Band structure features: the band gap size and the location of the direct band gap. Values for the minority spin component are given in brackets if they differ from the value for the majority spin component.

	band gap size [eV]	band gap location

FM	0.48	Γ
	(2.50)	(Γ)
SFM	0.50	Γ–X–Y plane
	(0.87)	(X and Y)
AF1	0.46	X and Y
AF2	0.92	Z
AF3	0.94	T

The valence and conduction bands (in the range [−1, +3] eV of the Fermi level) mainly have a V d-character, combined with a smaller fraction of O p-character, clearly showing these bands to originate from the VO_6_ chains of the MOF. For the valence band the band character is, more specifically, 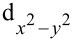 combined with a small fraction of p*_x_* and p*_y_* character of the O atoms in the planes of the octahedra. The lowest conduction bands, on the other hand, show complex combinations of different d-band characters combined with p-character of the O atoms. For the AF configurations the lowest conduction band at the band gap position always shows the same character makeup as the valence band. On the other hand, the second conduction band at these points is a mixture of 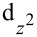, d*_xy_*, d*_xz_*, and d*_yz_* combined with p*_x_* and p*_y_* character of the O atoms in the vanadyl chains.

For all configurations, the valence and conduction bands along the high-symmetry lines split into two main groups: (1) For the high-symmetry lines parallel to the VO_6_ chains the bands show a clear dispersion. For the antiferromagnetic chains this dispersion is much smaller than for the ferromagnetic chains showing the repulsion between parallel unpaired V d-electrons in the 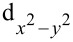 orbitals (cf. Figure 3). (2) The bands along the high-symmetry lines orthogonal to the VO_6_ chain direction, on the other hand, are extremely flat (with some exceptions, see below). As a result, the majority spin band gap for the FM configuration consists of two flat parallel bands covering the entire Γ–X–Y plane of the Brillouin zone. Upon closer examination, there is, however, a very small band splitting at the Γ-point for both the valence and the conduction band (about 20 meV in total) resulting in a direct band gap that is just marginally smaller than the band gap of the Γ–X–Y plane. This picture of dispersive bands parallel to a specific direction, and flat bands orthogonal to this direction is also found for other quasi-1D systems, such as atomic-scale nanowires [85]. This is another example of 1D behavior of the VO_6_ chains in MIL-47(V). Of the second group of high-symmetry lines, the Γ–Y and Z–T lines are also interesting to consider, since the zigzag of the vanadyl chain is located in this plane. Only for the antiferromagnetic chains, the valence and the conduction band show a finite dispersion, while flat bands are present for the ferromagnetic chains.

Combined, this shows that in the MIL-47(V) system, conductivity is expected to be directed almost entirely along the VO_6_ chains with the unpaired V d-electrons providing the current. The position of the direct band gap, depending on the spin configuration, makes this an interesting feature for experimental characterization, and validation of these results.

## Conclusion

In this work, the geometric and electronic structure of MIL-47(V) is investigated by using first principles calculations. An antiferromagnetic ground state is found, consisting of antiferromagnetic chains with an antiferromagnetic inter-chain coupling. This supports the experimental assumption of such a ground state favored over an antiferromagnetic ordering of ferromagnetic chains [1]. The derived coupling constants point toward an antiferromagnetic coupling between the chains, albeit two orders of magnitude weaker than the intra-chain coupling. The atomic structure of the different spin configurations is found to be nearly indistinguishable. However, the resulting small geometry based contribution to the system energy results in significant variations in the derived coupling constants.

The electronic band structure and the spin density distribution further confirm the quasi-1D nature of the VO_6_ chains in the MIL-47(V) MOF, with the conduction channel clearly located along the chain direction. Conduction and valence bands are found to exhibit almost perfectly flat bands along the high-symmetry lines orthogonal to the chains, which indicative of heavy-fermion behavior and reminiscent of the band structure of 1D systems.

The calculated bulk modulus and its pressure derivative show a clear relation between the spin configuration and the flexibility of the MIL-47(V) MOF, with antiferromagnetic chains increasing the bulk modulus significantly. By using the bulk modulus and its pressure derivative, the transition pressure for the large-pore-to-narrow-pore phase transition is derived and found to be in perfect agreement with experiments. The presence of 1D magnetic and electronic properties and the mechanic properties of the MIL-47(V) may provide interesting opportunities for sensing applications.

## Supporting Information

Supporting information contains the spin-dependent optimized MIL-47(V) structures. These structures have also been deposited in the Cambridge Crystalographic Data Center database CCDC 1021380–1021384.

File 1 MIL-47(V) structure in the FM spin configuration.

File 2 MIL-47(V) structure in the SFM spin configuration.

File 3 MIL-47(V) structure in the AF1 spin configuration.

File 4 MIL-47(V) structure in the AF2 spin configuration.

File 5 MIL-47(V) structure in the AF3 spin configuration.
